# The effects of preoperative glenohumeral osteoarthritis on rotator cuff repair: A systematic review and meta-analysis

**DOI:** 10.1371/journal.pone.0317560

**Published:** 2025-01-24

**Authors:** Sen Fang, Junwen Liang, Xudong Yang, Cairang Daoji, Zhixuan Nian, Mingchun Li, Jin Jiang, Xiangdong Yun

**Affiliations:** Department of Orthopaedics, Lanzhou University Second Hospital, Lanzhou, Gansu Province, China; Carol Davila University of Medicine and Pharmacy: Universitatea de Medicina si Farmacie Carol Davila din Bucuresti, ROMANIA

## Abstract

**Purpose:**

This meta-analysis was carried out to evaluate the clinical effectiveness of rotator cuff repair surgery in treating rotator cuff tears in individuals with mild glenohumeral osteoarthritis (GHOA).

**Methods:**

A computer-based search was conducted across multiple databases including PubMed, Embase, Web of Science, and Cochrane Library using the keywords "Shoulder Joints", "Osteoarthrosis", and "rotator cuff". Only studies focusing on patients with GHOA who underwent rotator cuff repair were considered for inclusion. The pertinent data was extracted and assessed for heterogeneity.

**Results:**

A total of 5 studies involving 924 patients were included in the meta-analysis. The treatment outcomes of patients with rotator cuff tears accompanied by mild GHOA and those with simple rotator cuff tears after rotator cuff repair were comparable in terms of retear(OR: 1.24; 95% CI 0.82–1.89; P = 0.31). The postoperative functional scores: the VAS score (MD: 0.14; 95% CI -0.19–0.47; P = 0.41)、ASES score (MD: -0.33; 95% CI -1.64–0.99)were similar between the two groups. Subgroup analysis of rotator cuff tears(small to moderate, MD: 0.85; 95%CI -0.65–2.39; p = 0.28; large to massive, MD: -1.94; 95% CI -8.45–4.58; P = 0.56), showed no difference in postoperative ASES scores between the two groups. Constant score (MD:-3.20; 95% CI -6.33–0.08; P = 0.04), external rotation (ER) in Range of motion (ROM) (MD: -4.42; 95% CI -6.72–2.13; P = 0.0002) and forward flexion (FF) in ROM(MD: -4.22; 95% CI -8.28–0.15; P = 0.04) were superior in patients with simple rotator cuff tears compared to those with rotator cuff tears accompanied by GHOA.

**Conclusion:**

Patients with rotator cuff tears and mild GHOA can achieve shoulder joint restoration after shoulder cuff repair surgery, and there is only a certain difference in postoperative Constant Score and ROM between these two groups.

**Trial registration:**

**PROSPERO registration** CRD42024565212.

## Introduction

With an incidence of 16.1% to 20.1%, glenohumeral osteoarthritis (GHOA) is a degenerative condition that primarily affects elderly people over the age of 65 [1]. According to studies, between 23 to 27% of patients with rotator cuff injuries also develop GHOA [2,3]. The relationship between rotator cuff injury and GHOA is complicated [4]. Several research studies indicate that rotator cuff tears are often considered a disadvantage for GHOA [5,6]. However, other research suggests that the existence of preoperative GHOA could also impact the results of rotator cuff repair procedures [7,8].

GHOA is an age-related disease. the current diagnosis of GHOA mainly relies on the imaging criteria of the Samilson and Prieto classification [5,9–11], and GHOA is divided into four grades. grades: 0 = normal, grades:1 = mild (osteophytes smaller than 3 mm on the humeral head), grades:2 = moderate (osteophytes between 3 and 7 mm on the humeral head or the glenoid rim), or grades:3 = severe (osteophytes larger than 7 mm, with or without articular incongruity). Another method of comparison is the modified Kellgren—Lawrence (K—L) classification, which is divided into five levels [12]. K-L: 0:mild, K-L-Ⅰand above. Both classifications are based on imaging. For patients with severe OA and rotator cuff tears, some scholars have adopted reverse total shoulder arthroplasty or anatomical total shoulder arthroplasty, and the results show that both procedures have a positive impact on the quality of life of patients [13,14]. Since many cases of GHOA are mild to moderate, when they accompany a torn rotator cuff, they can frequently be treated with rotator cuff repair alone [15,16].

The management of rotator cuff tears in clinical settings may vary depending on the extent of the injury, encompassing conservative approaches as well as surgical interventions [17]. While rotator cuff repairs have demonstrated favorable long-term outcomes in the broader population, their structural and functional effectiveness in individuals with concomitant GHOA has been sparsely investigated in the existing literature. Therefore, the aim of this research was to assess the impact of preoperative rotator cuff tears in conjunction with mild GHOA on the outcomes of rotator cuff repair surgery.

## Materials and methods

This meta-analysis was conducted and reported in compliance with the guidelines of the preferred reporting items for systematic reviews and meta-analyses [18], and it was registered in the international prospective register of systematic reviews (CRD42024565212). All analyses were based on previously published research; Therefore, ethical approval and patient consent are not required.

### Search strategy and study selection

At the planning stage of the search, based on the Cochrane Collaboration’s recommendations, the search method was created (S2 Table).

An electronic database search was conducted on titles and abstracts from inception to December 1, 2024 using the following databases: PubMed, Embase, Web of Science, and the Cochrane Library. For the keywords search strategy used see Table 1.

**Table 1:** Search strategy.

	Search Term(eg, PubMed)
1	(Shoulder Joints [Titel/Abstract] OR Glenohumeral Joint [Titel/Abstract] OR Glenohumeral Joints [Titel/Abstract] OR Glenoid Labrum [Titel/ Abstract]) OR (“Shoulder Joints” [Mesh])
2	(Osteoarthritides [Titel/Abstract] OR Osteoarthrosis [Titel/Abstract] OR Osteoarthroses [Titel/Abstract] OR Degenerative Arthritides [Titel/Abstract] OR Degenerative Arthritis [Titel/Abstract] OR Arthrosis [Titel/Abstract] OR Arthroses [Titel/Abstract] OR Osteoarthrosis Deformans [Titel/Abstract]) OR (“Osteoarthrosis” [Mesh])
3	(rotator cuff [Titel/ Abstract] OR Rotator Cuffs [Titel/ Abstract]) OR (“rotator cuff” [Mesh])
4	1 AND 2 AND 3 limited to English language

The search was carried out utilizing MeSH and Emtree terms in conjunction with free text. Furthermore, additional relevant literature was sought by examining the references of the included studies and reviews.

### Inclusion and exclusion criteria

The meta-analysis only included studies that fulfilled the following requirements: (1) The case group consisted of patients with a primary rotator cuff tear diagnosed by computed tomography (CT) or Magnetic resonance imaging (MRI) with mild GHOA(K-L classification: grade 0 or the Samilson and Prieto classification:grade 1); (2) The control group was comprised of patients only clinically diagnosed with rotator cuff tears requiring rotator cuff repair; (3) Participants were at least 18 years old, regardless of gender or race; (4) The follow-up period was at least 12 months, and (5) Literature with at least one of the following indicators: retear rate, Constant score, visual analog scale (VAS), American Shoulder and Elbow Surgeons Score (ASES), Range of motion (ROM), containing forward flexion (FF) and external rotation (ER). The following were excluded from the meta-analysis: (1) Studies in which it was impossible to extract or convert valid data; (2) Animal or cadaveric studies; (3) Republished literature, and (4) Poor quality literature.

### Review process

Two reviewers conducted a thorough examination of the literature, collected data, and performed validation checks following the guidelines outlined in the Preferred Reporting Items for Systematic Reviews and Meta-analyses (PRISMA) methodology [19]. In cases of disagreement between the two researchers, a third scholar was consulted for resolution.

### Data extraction

The titles and abstracts of all papers were scrutinized by two separate reviewers to determine the selection of articles for full-text evaluation. Information regarding the authors, publication date, sample size, level of evidence, patient characteristics, research methodology, specific type of rotator cuff tear, surgical techniques, and diagnostic approach for GHOA was collected from the selected studies. In the event that these data were not provided, the research in question were disqualified from further consideration.

### Quality assessment

To evaluate and rank the methodological quality of the included research, we utilized the Methodological Index for Non-Randomized Studies (MINORS) score [20]. The MINORS was used to assess the quality of each study and is considered a strong quality assessment tool for studies of this nature, whereas assessment of the risk of bias was completed independently by two authors. A third author resolved any discrepancies. Seven items were selected for assessment of non-comparative studies and five for use with comparative studies in MINORS. A priori categories for methodological quality were as follows: for non-comparative and comparative studies, a score of 0–8 or 0–12 was deemed low, a score of 9–12 or 13–18 was deemed intermediate, and a score of 13–16 or 19–24 was deemed high.

### Level of evidence

The Grading of Recommendations Assessment, Development, and Evaluation (GRADE) framework was employed to assess the overall certainty of the evidence [21]. The evidence was categorized into levels of high, moderate, low, or very low certainty. Two reviewers conducted independent assessments of the GRADE outcomes, with a third reviewer resolving any discrepancies that arose.

### Statistical analysis

Use of Review Manager (RevMan) Version 5.4 was made for statistical analysis. To determine heterogeneity, the chi-squared test was performed. If the value of *I*^*2*^ was greater than 50%, a random-effects model was applied; otherwise, a fixed-effects model was applied. For the pooled analysis of continuous variables, weighted mean differences (MD) were employed in place of odds risk (OR) for dichotomous variables. For each variable, estimates of the 95% confidence interval (CI) and the outcomes of the hypothesis test were listed in a forest plot. The same method was applied for the subgroup analysis. A sensitivity analysis was conducted to exclude studies exhibiting a significant risk of bias.

## Results

### Search results

**Fig 1:**
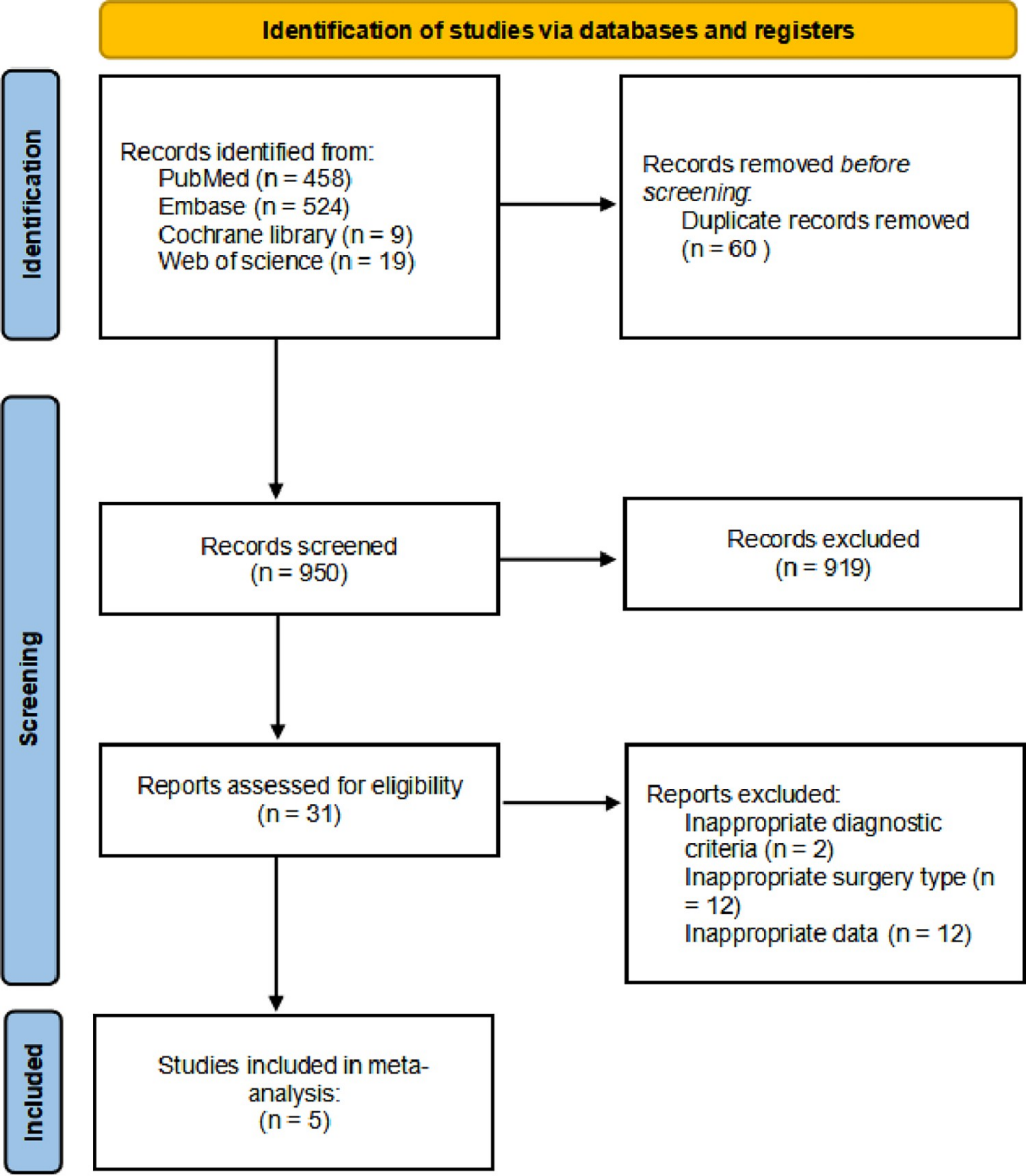
Search result.

The literature search together with cross-referencing yielded 950 articles after duplicates were removed. Finally, 31 articles were selected for full-text review followed by exclusion of 26 articles as they failed to meet our inclusion criteria (S1 Table). The last 5 studies, involving 924 patients, were included [5,9–12] (Fig 1).

### Characteristics of included studies

There were 561 patients in the GHOA group and 363 patients in the control group. The follow-up period ranged at least 12 months. One of the studies that were considered for inclusion was carried out in Europe (1 in Finland), two were carried out in Asia (2 in Korea), and two were carried out in North America (2 in the United States). The articles had publication years ranging from 2014 to 2024. The 5 studies that were included were all retrospective comparative studies. The majority of the patients who underwent rotator cuff repair were middle-aged and older individuals with ages ranging from 30 to 80. Imaging techniques were used to identify and assess GHOA. The main defining characteristics for the mentioned studies are summarized in Table 2.

**Table 2:** Study characteristics.

References	Location	Total participant	Age (years)	Gender (male%)	Follow-up (month)	Diagnostic method of GHOA	GHOA classionfication	Study design	Study design (level of evidence)
Reddy 2022 [11]	USA	206	>30	47.1	≥12	MRI	the modified Kellgren–Lawrence (K–L) classification	Retrospective comparative study	Level III; Retrospective Cohort Comparison; Treatment Study
Hong 2022 [9]	USA	142	NR	41	≥24	MRI	the modified Samilson and Prieto classification	Retrospective comparative study	Level Ⅱ; Retrospective Cohort Design; Treatment Study
Kim 2021 [5]	Korea	348	>60	42.2	≥12	MRI	the Samilson and Prieto method	Retrospective comparative study	Level III; Retrospective Cohort Comparison; Treatment Study
Jeong 2018	Korea	146	41–79	43.2	≥24	MRI	the Samilson-Prieto Classification	Retrospective comparative study	Level III; Retrospective Cohort Design; Treatment Study
Kukkonen 2013 [12]	Finland	82	>57.1	NR	12	Radiographs	the Samilson-Prieto Classification	Retrospective comparative study	level III, Clinical retrospective comparative registry study

NR: Not reporte.

### Quality assessment

Of the 5 articles included, 4 articles were retrospective comparative study classified as Level III evidence and and1 was Level II retrospective comparative study. Based on the MINORS criteria (Table 3), the mean study quality score was 17.2 ± 1.6. These results indicated that the literature included in the study was good, which enhances the credibility of the conclusions drawn from our meta-analysis.

**Table 3:** Quality assessment of included studies.

References	Clearly stated aim	Inclusion of consecutive patient	Prospective collection of data	Endpoints appropriate for aim	Unbiased assessment of endpoints	Follow-up period appropriate follow-up period	Lost to follow-up < 5%	Prospective calculation of study size	Adequate control group	Contem-porary groups	Baseline equivalence of groups	Adequate statistical analysis	Total score
Reddy 2022 [11]	2	1	1	2	0	2	0	0	2	2	2	2	16
Hong 2022 [9]	2	2	0	2	0	2	0	0	2	2	2	2	16
Kim 2021 [5]	2	2	2	2	0	2	0	0	2	2	2	2	18
Jeong 2018	2	2	2	2	0	2	2	0	2	2	2	2	20
Kukkonen 2013 [12]	2	2	2	2	0	2	0	0	2	2	0	2	16

### Outcomes

#### Retear rate

**Fig 2:**
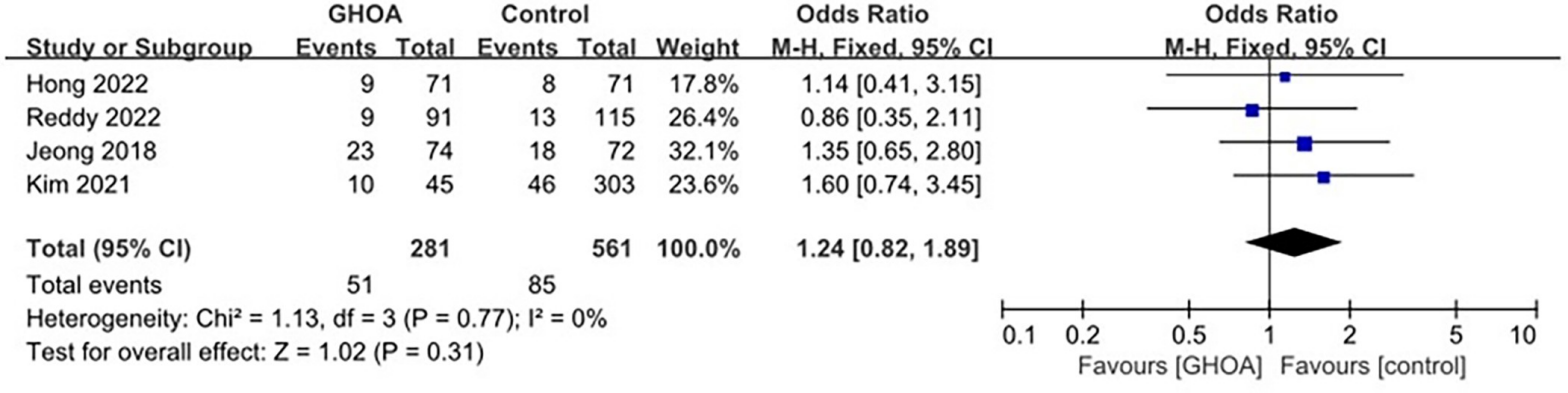
Forest plot of retear rate.

There were 105 patients in the control group and 110 patients in the GHOA group in the four studies that examined the retear rate [5,9–11]. Four studies were followed up for at least 12 months, and all studies used MRI for postoperative evaluation. No heterogeneity was observed among the studies, (*I*^*2*^ = 0, P = 0.53), therefore the fixed-effects model was selected. The incidence of rotator cuff retear after surgery was similar in both the case group and the control group with rotator cuff tear and GHOA (OR: 1.24; 95% CI 0.82–1.89; P = 0.31, Fig 2).

#### VAS score

**Fig 3:**
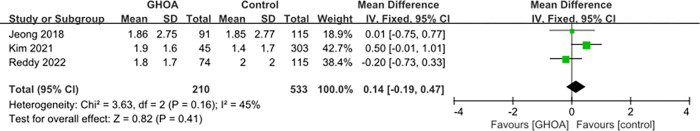
Forest plot of the VAS score.

A total of three studies [5,10,11], including 760 patients, was used to assess whether there were differences in the VAS score between the GHOA and control groups. The fixed-effects model was chosen because the degree of homogeneity across all of the studies was satisfactory (*I*^*2*^ = 0, P = 0.40). During the span of at least one year of subsequent observation, there was not a statistical difference in the VAS scores of the control group and the case group with rotator cuff injury with GHOA (MD: 0.14; 95% CI -0.19–0.47; P = 0.41; Fig 3).

#### ASES

**Fig 4:**
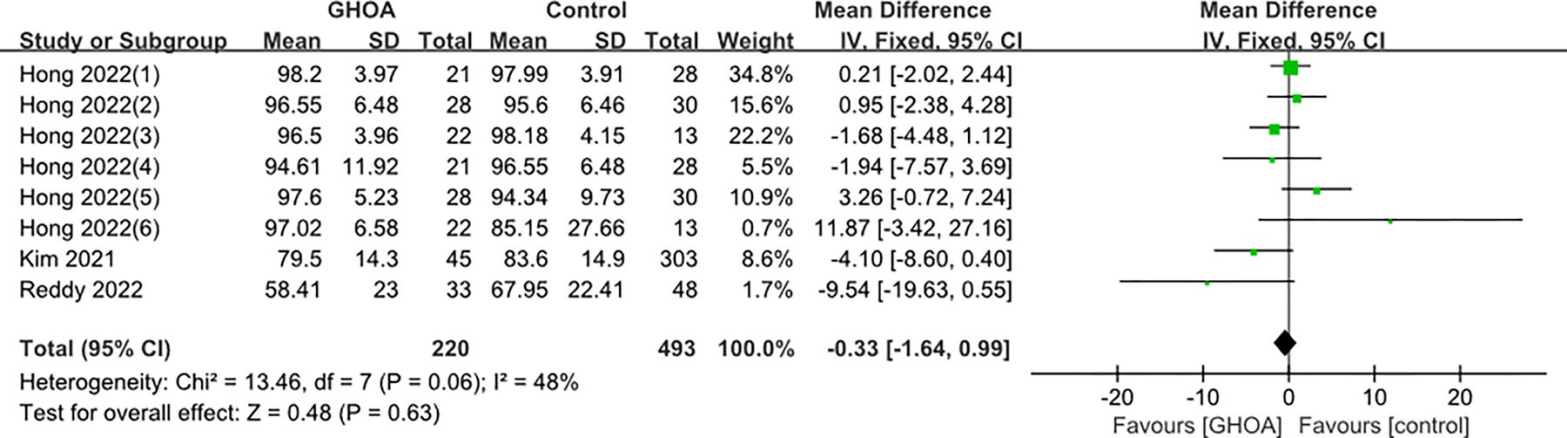
Forest plot of the ASES. (Hong 2022 (1): Shoulder right/ small tear; Hong 2022(2): Shoulder right/ medium tear; Hong 2022(3): Shoulder right/ large tear; Hong 2022(4): Shoulder left/ small tear; Hong 2022(5): Shoulder left/ medium tear; Hong 2022(6): Shoulder right/ large tear).

**Fig 5:**
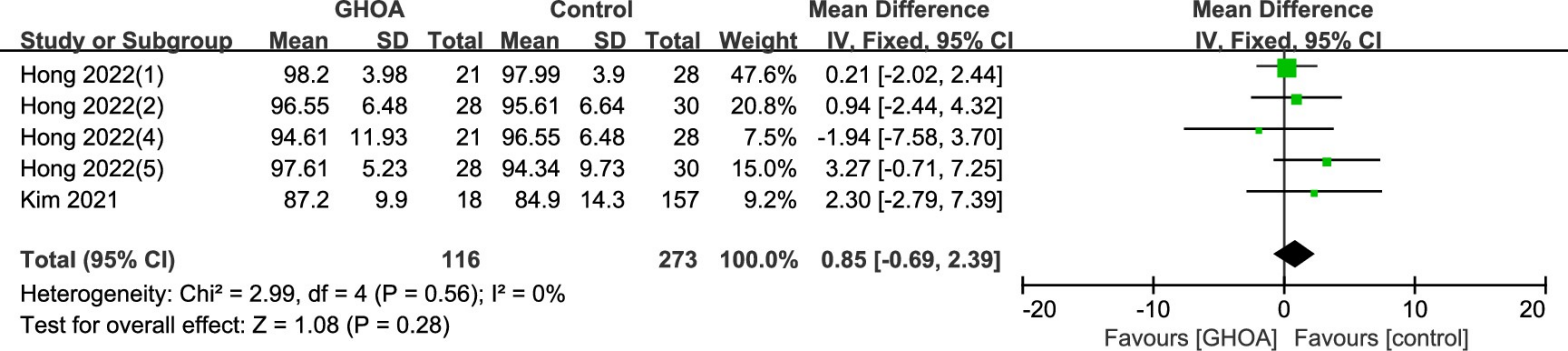
Forest plot of the subgroup of ASES on small-to-medium. (Hong 2022(1): Shoulder right/ small tear; Hong 2022(2): Shoulder right/ medium tear; Hong 2022(4): Shoulder left/ small tear; Hong 2022(5): Shoulder left/ medium tear).

**Fig 6:**
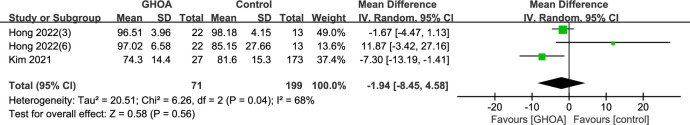
Forest plot of the subgroup of ASES on large-to-massive (Hong 2022 (3): Shoulder right/ large tear; Hong 2022 (6): Shoulder right/ large tear).

A total of three studies were included [5,9,11], including 713 subjects. A detailed group analysis was carried out in Hong’s study [9] since both of the participants’ shoulders were included in the study index and rotator cuff injuries were categorized into three degrees (small, moderate, and big tears). According to the findings of a meta-analysis using a fixed-effects model, there was not a statistically significant difference in the postoperative ASES ratings of patients who had rotator cuff tears and GHOA and those who were in the control group. (MD: -0.33; 95% CI -1.64–0.99; P = 0.63; Fig 4). Given the varying degrees of rotator cuff tears, a subgroup analysis was conducted in this study. Participants were categorized into two groups based on the size of their tears: small to moderate tears (MD: 0.85; 95%CI -0.65–2.39; P = 0.28; Fig 5) and large to massive tears (MD: -1.94; 95% CI -8.45–4.58; P = 0.56; Fig 6). Due to the large heterogeneity of large-to-massive tear degree, a random effects model is chosen. However, the subgroup analysis did not have a significant impact on the results.

#### Constant score

**Fig 7:**
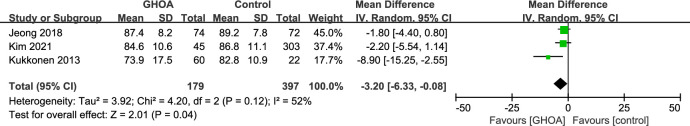
Forest plot of the constant score.

There was a total of 399 patients in the GHOA group and 177 patients in the control group who were included in the 3 studies that reported on the Constant score [5,10,12]. The included studies did not show significant heterogeneity(*I*^*2*^ = 52%). The follow-up period for the three studies was a minimum of 12 months, and a significant correlation was observed between the Constant score and the GHOA group. It was found that shoulder cuff repair can lead to improvements in shoulder joint function among patients with GHOA (MD: -3.20; 95% CI -6.33–0.08; P = 0.04; Fig 7).

#### ROM

**Fig 8:**
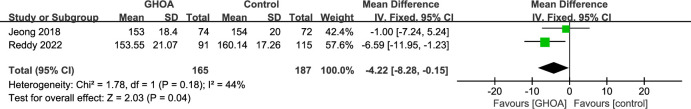
Forest plot of FF.

**Fig 9:**
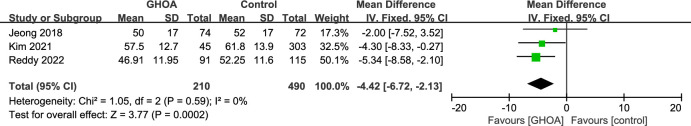
Forest plot of ER.

The shoulder joint’s range of motion encompasses both FF and ER positions. In 2 publications with 352 subjects, FF parameters were used [10,11]. According to the findings of a meta-analysis that used a fixed effect model, there was a statistically significant difference in postoperative FF between the group of patients with rotator cuff tears who had GHOA and the control group (MD: -4.22; 95% CI -8.28–0.15; P = 0.04; Fig 8). There were 700 participants with ER parameters in 3 literatures [5,10,11]. The included studies had low heterogeneity using a fixed-effect model (*I*^*2*^ = 0). Rotator cuff tear with GHOA was significantly correlated with ER (MD: -4.42; 95% CI -6.72–2.13; P = 0.0002; Fig 9).

#### GRADE outcomes

According to the GRADE approach, the retear rate, VAS, ASES score, Constant score, and range of motion in FF and ER were graded as moderate (Table 4).

**Table 4:** Overall evidence quality according to the Grading of Recommendations Assessment, Development and Evaluation (GRADE) approach.

	Number of studies	Study design	Certainty assessment					Effect of estimate OR/(S)MD(95%CI)	Certainty
			Risk of bias	Inconsistency	Indirectness	Imprecision	Publication bias		
Retear rate	4	Observational studies	Not serious	Not serious	Not serious	Not serious	None	OR 1.24 (0.82 to 1.89)	⨁⨁⨁◯ Moderate
VAS score	3	Observational studies	Not serious	Not serious	Not serious	Not serious	None	MD 0.14 higher (0.19 lower to 0.47 higher)	⨁⨁⨁◯ Moderate
ASES	8	Observational studies	Not serious	Not serious	Not serious	Not serious	None	MD 0.33 lower (1.64 lower to 0.99 higher)	⨁⨁⨁◯ Moderate
ASES small-Medium	5	Observational studies	Not serious	Not serious	Not serious	Not serious	None	MD 0.85 higher (0.69 lower to 2.39 higher)	⨁⨁⨁◯ Moderate
ASES Large-Massive	3	Observational studies	Not serious	Not serious	Not serious	Not serious	None	MD 1.94 lower (8.45 lower to 4.58 higher)	⨁⨁⨁◯ Moderate
Constant score	3	Observational studies	Not serious	Not serious	Not serious	Not serious	None	MD 3.2 lower (6.33 lower to 0.08 lower)	⨁⨁⨁◯ Moderate
ROM(FF)	2	Observational studies	Not serious	Not serious	Not serious	Not serious	None	MD 4.22 lower (8.28 lower to 0.15 lower)	⨁⨁⨁◯ Moderate
ROM(ER)	3	Observational studies	Not serious	Not serious	Not serious	Not serious	None	MD 4.42 lower (6.72 lower to 2.13 lower)	⨁⨁⨁◯ Moderate

## Discussion

In this meta-analysis, the impact of mild GHOA on the treatment of rotator cuff tears during repair showed significant differences in Constant score and range of motion in FF and ER. However, there were no variations in retear rate, VAS score, and ASES score.

There is currently no established standard for the clinical management of rotator cuff injury with GHOA [22]. Whether by conservative therapy [23], arthroscopic surgery [24], open surgery, or shoulder replacement, the goal is to enhance shoulder movement and functional rehabilitation. Different degrees of GHOA necessitate varied treatment approaches; therefore, the clinical surgeon must meticulously select the most suitable surgical technique based on the severity and specific location of the patient’s injury.

It is essential to have a thorough understanding of the enduring effects of GHOA on the results of rotator cuff repair. The results of our statistical comparison of the GHOA group and the control group revealed there was no discernible difference in the rate of retears between the two groups. For the management of rotator cuff tears, the retear rate is a crucial outcome indicator. Despite the considerable progress made in surgical methods for repairing rotator cuff injuries, orthopedic surgeons continue to be concerned about the occurrence of retears following the procedure [25]. According to research conducted in the past, the postoperative retear rate might range anywhere from 36 to 94% [26]. The retear rates in Jeong’s study were 31.1% in the OA group and 25.0% in the control group, there was not a clear difference in the rate of unsuccessful repair attempts between the two groups [10]. This is consistent with the results of our meta-analysis. In the group of patients who also have GHOA in addition to rotator cuff rupture, excellent outcomes for rotator cuff repair are anticipated in terms of postoperative outcomes and OA progression. But the temporal variations may arise from the use of different evaluation criteria for retear rates in studies, as well as the higher diagnostic accuracy of MRI scans taken after surgery compared to those taken beforehand [5,10,11]. This fact indicates that long-term clinical follow-up is still required.

The ultimate goal of rotator cuff injury treatment is to recover shoulder function, this meta-analysis concluded that rotator cuff repair is equally effective in treating patients with mild GHOA and rotator cuff tears in the early stages. There were no statistically significant differences in the clinical outcomes or the ASES, VAS preoperatively or at the end of the follow-up period between the group with GHOA and the group without GHOA. ASES are significant indications for assessing shoulder joint function. ASES has a better response effect in the assessment of shoulder joint function [27]. Because the size of the rotator cuff injury has an impact on the postoperative shoulder joint function score [28], based on existing data, this study conducted a statistical analysis based on the size of the rotator cuff tear in order to reduce the interference of the rotator cuff injury size on statistical results. The ASES scores reported in the literature were divided into two categories: small to large tear groups and large to enormous tear groups. Based on our subgroup study, which demonstrates similar and favorable outcomes in postoperative shoulder function recovery as patients with mild GHOA, rotator cuff prosthesis is a reasonable therapy alternative for these patients.

The management of shoulder joint pain following rotator cuff repair is crucial. From the point of view of pathogenesis, immunochemistry has shown torn rotator cuff tendons with lower vascularity have fewer new nerve fibers and is linked to lower chronic pain [29]. This is consistent with our clinical observation. Since GHOA is also the cause of shoulder pain, this partly explains why VAS score has no statistical difference. For individuals with rotator cuff damage and mild GHOA, there is cause to believe that standard rotator cuff repair will result in a successful recovery.

Our analysis results showed that Constant score and ROM were statistically different between the two groups. Patients with rotator cuff tears with GHOA had lower mean scores on Constant score and ROM than those with rotator cuff tears alone. This is consistent with previous findings [7]. Glenohumeral instability due to rotator cuff tears may affect clinical outcomes in patients with mild to moderate OA. Especially in patients with large rotator cuff tears, osteoarthritis of the shoulder joint can disrupt the balance of strength in the shoulder, resulting in shoulder instability [30].

The more severe the GHOA, the worse the RCR outcome, therefore, early diagnosis and interventional treatment of patients with GHOA must be initiated as early as possible. The glenohumeral joint, unlike the weight-bearing knee and hip joints, does not experience severe clinical impairment in mild osteoarthritis due to its non-weight-bearing nature. Based on the results of our meta-analysis, our study focused on the core idea that patients with rotator cuff tears with mild to moderate osteoarthritis did not differ significantly in postoperative retear rates, major functional recovery, and pain measures compared with patients with rotator cuff tears alone. This suggests that we should pay attention to and solve the problem of rotator cuff injury during surgery. However, we still recommend long-term follow-up of patients with mild GHOA in order to more fully evaluate the progression and treatment effect. In a cohort study, Chalmers et al. [31] found that the progression of glenohumeral arthritic changes resulting from degenerative cuff disease remains minimal over an 8-year period, regardless of the severity or size of tears at midterm time points. There is debate surrounding the available treatments for mild GHOA [22]. In the study of Kukkonen et al. [12] surgical treatment was no better than conservative treatment for patients over 55 years of age with small, non-traumatic, single-tendon supraspinatus tears. However, Hill’s study [32] showed modest improvement in postoperative pain and functional scores at least 1 year of follow-up in a group of patients receiving RCR with humeral glenohumeral microfractures. Consequently, it is imperative to examine the efficacy of rotator cuff restoration in individuals who have minor GHOA-related rotator cuff injuries.

Our study had a few limitations. First, because the five studies included in this investigation were all retrospective, their statistical power was lower than that of clinical randomized controlled trials. Furthermore, with only five research included, we were unable to construct a funnel plot to measure publication bias. As a result, the study’s conclusions need to be supplemented and verified by further relevant studies in order to be more comprehensive and reliable. Furthermore, there were differences in data collection methods and inclusion/exclusion criteria among the studies, which may have an impact on the results of this study and may introduce heterogeneity due to differences in study design, thus requiring careful consideration of these potential factors when interpreting and applying the study’s findings.

Second, to address the diversity of GHOA classification, this study included five investigations, one utilizing the K-L classification method and the others using the Samilson and Prieto classification method. In the study, the K-L classification method was only used in the summary analysis of the Constant scoring system, and the results showed that its heterogeneity was not significant, and its weight in the analysis was only 17.7%. As a result, we believe these findings retain some relevance. To improve the study’s accuracy and dependability, future research can undertake comparative analyses using the same baseline level and uniform classification standards. Such a research design will aid in reaching more consistent and convincing conclusions. Furthermore, because GHOA is classified as a chronic disease, the long-term impacts and results require further confirmation and investigation through a significant number of extra investigations.

Finally, while this study attempted to include as many English literatures as possible, it does have a limitation in that it does not cover a variety of studies from different countries or studies involving different ethnic groups, limiting the universality and wide applicability of the findings. Taking into account the features of individual exercise groups, these elements may become potential confounding factors, affecting the study’s results significantly. As a result, in order to gain a more comprehensive knowledge and increase the study’s universality, future research should seek to include more diverse samples, including other ethnic groups and sports groups, to further validate and broaden the study’s conclusions.

## Conclusion

The presence of mild GHOA prior to surgery has minimal impact on shoulder cuff repair, as evidenced by a statistical difference in the Constant Score between patients with GHOA and those with simple rotator cuff tears, but without clinical significance.

## Supporting information

S1 Checklist PRISMA 2020 checklist. (DOCX)

S1 Table Reports excluded. (DOCX)

S2 Table List of abbreviations. (DOCX)

S1 Dataset (DOCX)
