# Supplementary material for: The effects of preoperative glenohumeral osteoarthritis on rotator cuff repair: A systematic review and meta-analysis
Source: PLoS One. 2025 Jan 24;20(1):e0317560. doi: 10.1371/journal.pone.0317560 (PMC11759359; doi:10.1371/journal.pone.0317560)
Supplement: S1 Table — (DOCX) [file pone.0317560.s002.docx]

**S1 Table Reports excluded**

| **Article** | **numerical order** | **Reason for exclusion** |
| --- | --- | --- |
| Wright 2024 | 1 | Inappropriate data |
| Tokgöz 2023 | 2 | Inappropriate data |
| Jennewine 2024 | 3 | Inappropriate data |
| Yiannakopoulos 2021 | 4 | Inappropriate surgery type |
| Simone 2014 | 5 | Inappropriate surgery type |
| Razmjou 2015 | 6 | Inappropriate diagnostic criteria |
| Raiss 2014 | 7 | Inappropriate surgery type |
| Puzzitiello 2021 | 8 | Inappropriate surgery type |
| Plachel 2019 | 9 | Inappropriate data |
| Ozel 2020 | 10 | Inappropriate surgery type |
| Mizuno 2013 | 11 | Inappropriate surgery type |
| Matsuba 2018 | 12 | Inappropriate data |
| Mahony 2018 | 13 | Inappropriate data |
| Liu 2022 | 14 | Inappropriate surgery type |
| Larsen 2022 | 15 | Inappropriate data |
| Kim 2011 | 16 | Inappropriate diagnostic criteria |
| Iriberri 2015 | 17 | Inappropriate surgery type |
| Hill 2021 | 18 | Inappropriate surgery type |
| Herve 2019 | 19 | Inappropriate data |
| Heifner 2021 | 20 | Inappropriate data |
| Hattrup 2012 | 21 | Inappropriate surgery type |
| Gbejuade 2022 | 22 | Inappropriate data |
| Flurin 2017 | 23 | Inappropriate data |
| Compagnoni 2019 | 24 | Inappropriate data |
| Chalmers 2019 | 25 | Inappropriate surgery type |
| Ahearn 2013 | 26 | Inappropriate surgery type |

[1-26]

1. Wright MA, Smith MJ, Roach CJ (2024) Treatment of Shoulder Osteoarthritis With Intact Rotator Cuff and Severe Glenoid Retroversion. J Am Acad Orthop Surg 32:e737-e740. DOI 10.5435/jaaos-d-23-00669

2. Tokgöz MA, Elma T, Yapar A, Özer M, Ataoğlu MB, Kanatli U (2023) Does the presence of arthroscopically detected stage 1-2 glenohumeral osteoarthritis have any clinical impact on the outcome of arthroscopic rotator cuff repairs? Turk J Med Sci 53:218-224. DOI 10.55730/1300-0144.5576

3. Jennewine BR, James NF, Polio WP, Naser AM, Nieboer MJ, Schoch BS, Throckmorton TW, Bernholt DL, Azar FM, Brolin TJ (2024) Superior humeral head osteophytes are associated with rotator cuff insufficiency in glenohumeral osteoarthritis: a retrospective analysis. Eur J Orthop Surg Traumatol 34:893-900. DOI 10.1007/s00590-023-03727-3

4. Yiannakopoulos CK, Vlastos I, Theotokatos G, Galanis N (2021) Acromioclavicular joint arthritis is not an indication for routine distal clavicle excision in arthroscopic rotator cuff repair. Knee Surg Sports Traumatol Arthrosc 29:2090-2095. DOI 10.1007/s00167-020-06098-y

5. Simone JP, Streubel PH, Sperling JW, Schleck CD, Cofield RH, Athwal GS (2014) Anatomical total shoulder replacement with rotator cuff repair for osteoarthritis of the shoulder. Bone Joint J 96-b:224-228. DOI 10.1302/0301-620x.96b.32890

6. Razmjou H, ElMaraghy A, Dwyer T, Fournier-Gosselin S, Devereaux M, Holtby R (2015) Outcome of distal clavicle resection in patients with acromioclavicular joint osteoarthritis and full-thickness rotator cuff tear. Knee Surg Sports Traumatol Arthrosc 23:585-590. DOI 10.1007/s00167-014-3114-2

7. Raiss P, Zeifang F, Pons-Villanueva J, Smithers CJ, Loew M, Walch G (2014) Reverse arthroplasty for osteoarthritis and rotator cuff deficiency after previous surgery for recurrent anterior shoulder instability. Int Orthop 38:1407-1413. DOI 10.1007/s00264-014-2325-y

8. Puzzitiello RN, Moverman MA, Menendez ME, Hart PA, Kirsch J, Jawa A (2021) Rotator cuff fatty infiltration and muscle atrophy do not impact clinical outcomes after reverse total shoulder arthroplasty for glenohumeral osteoarthritis with intact rotator cuff. J Shoulder Elbow Surg 30:2506-2513. DOI 10.1016/j.jse.2021.03.135

9. Plachel F, Korn G, Ortmaier R, Hoffelner T, Resch H, Moroder P (2019) Repair failure increases the risk of developing secondary glenohumeral osteoarthritis: A long-term follow-up after open repair of large subscapularis tendon tears. Orthop Traumatol Surg Res 105:1529-1533. DOI 10.1016/j.otsr.2019.09.021

10. Ozel O, Hudek R, Abdrabou MS, Werner BS, Gohlke F (2020) The implications of the glenoid angles and rotator cuff status in patients with osteoarthritis undergoing shoulder arthroplasty. BMC Musculoskelet Disord 21:668. DOI 10.1186/s12891-020-03690-8

11. Mizuno N, Denard PJ, Raiss P, Walch G (2013) Reverse total shoulder arthroplasty for primary glenohumeral osteoarthritis in patients with a biconcave glenoid. J Bone Joint Surg Am 95:1297-1304. DOI 10.2106/jbjs.L.00820

12. Matsuba T, Hata Y, Ishigaki N, Nakamura K, Kato H (2018) Osteoarthritis progression of the shoulder: A long-term follow-up after mini-open rotator cuff repair. J Orthop Surg (Hong Kong) 26:2309499018768106. DOI 10.1177/2309499018768106

13. Mahony GT, Werner BC, Chang B, Grawe BM, Taylor SA, Craig EV, Warren RF, Dines DM, Gulotta LV (2018) Risk factors for failing to achieve improvement after anatomic total shoulder arthroplasty for glenohumeral osteoarthritis. J Shoulder Elbow Surg 27:968-975. DOI 10.1016/j.jse.2017.12.018

14. Liu H, Huang TC, Yu H, Wang Y, Wang D, Long Z (2022) Total shoulder arthroplasty versus reverse total shoulder arthroplasty: Outcome comparison in osteoarthritis patients with or without concurrent rotator cuff deficiency. Medicine (Baltimore) 101:e29896. DOI 10.1097/md.0000000000029896

15. Larsen JB, Østergaard HK, Thillemann TM, Falstie-Jensen T, Reimer LCU, Noe S, Jensen SL, Mechlenburg I (2022) Are progressive shoulder exercises feasible in patients with glenohumeral osteoarthritis or rotator cuff tear arthropathy? Pilot Feasibility Stud 8:168. DOI 10.1186/s40814-022-01127-8

16. Kim J, Chung J, Ok H (2011) Asymptomatic acromioclavicular joint arthritis in arthroscopic rotator cuff tendon repair: a prospective randomized comparison study. Arch Orthop Trauma Surg 131:363-369. DOI 10.1007/s00402-010-1216-y

17. Iriberri I, Candrian C, Freehill MT, Raiss P, Boileau P, Walch G (2015) Anatomic shoulder replacement for primary osteoarthritis in patients over 80 years: outcome is as good as in younger patients. Acta Orthop 86:298-302. DOI 10.3109/17453674.2015.1006036

18. Hill BW, Singh AM, Astolfi M, Horneff JG, Schoch BS, Abboud JA (2021) Outcomes of rotator cuff repair with concurrent microfracture of focal glenohumeral osteoarthritis. J Shoulder Elbow Surg 30:S66-s70. DOI 10.1016/j.jse.2021.04.008

19. Herve A, Thomazeau H, Favard L, Colmar M, Mansat P, Walch G, Betz M, Kempf JF, Collin P (2019) Clinical and radiological outcomes of osteoarthritis twenty years after rotator cuff repair. Orthop Traumatol Surg Res 105:813-818. DOI 10.1016/j.otsr.2019.02.013

20. Heifner JJ, Kumar AD, Wagner ER (2021) Glenohumeral osteoarthritis with intact rotator cuff treated with reverse shoulder arthroplasty: a systematic review. J Shoulder Elbow Surg 30:2895-2903. DOI 10.1016/j.jse.2021.06.010

21. Hattrup SJ, Sanchez-Sotelo J, Sperling JW, Cofield RH (2012) Reverse shoulder replacement for patients with inflammatory arthritis. J Hand Surg Am 37:1888-1894. DOI 10.1016/j.jhsa.2012.05.015

22. Gbejuade H, Patel MS, Singh H, Modi A (2022) Reconstruction of irreparable rotator cuff tears with an acellular dermal matrix in elderly patients without joint arthritis. Shoulder Elbow 14:83-89. DOI 10.1177/1758573220965535

23. Flurin PH, Hardy P, Valenti P, Meyer N, Collin P, Kempf JF (2017) Osteoarthritis after rotator cuff repair: A 10-year follow-up study. Orthop Traumatol Surg Res 103:477-481. DOI 10.1016/j.otsr.2017.03.007

24. Compagnoni R, Stoppani C, Menon A, Cosmelli N, Fossati C, Ranuccio F, Randelli P (2019) Management of Acromioclavicular Osteoarthritis in Rotator Cuff Tears: A Scoping Review. Joints 7:199-204. DOI 10.1055/s-0041-1730378

25. Chalmers PN, Granger E, Ross H, Burks RT, Tashjian RZ (2019) Preoperative Factors Associated With Subsequent Distal Clavicle Resection After Rotator Cuff Repair. Orthop J Sports Med 7:2325967119844295. DOI 10.1177/2325967119844295

26. Ahearn N, McCann PA, Tasker A, Sarangi PP (2013) The influence of rotator cuff pathology on functional outcome in total shoulder replacement. Int J Shoulder Surg 7:127-131. DOI 10.4103/0973-6042.123509
