# Supplementary material for: The effects of preoperative glenohumeral osteoarthritis on rotator cuff repair: A systematic review and meta-analysis
Source: PLoS One. 2025 Jan 24;20(1):e0317560. doi: 10.1371/journal.pone.0317560 (PMC11759359; doi:10.1371/journal.pone.0317560)
Supplement: S2 Table — (DOCX) [file pone.0317560.s003.docx]

| List of abbreviations | |
| --- | --- |
| GHOA | glenohumeral osteoarthritis |
| ASES | American Shoulder and Elbow Surgeons Score |
| VAS | visual analog scale |
| ROM | range of motion |
| ER | external rotation |
| FF | forward flexion |
| MD | mean differences |
| CI | confidence interval |
| OR | odds risk |
| GRADE | Grading of Recommendations Assessment, Development, and Evaluation |
| RevMan | Review Manager |
