# Supplementary material for: The effects of preoperative glenohumeral osteoarthritis on rotator cuff repair: A systematic review and meta-analysis
Source: PLoS One. 2025 Jan 24;20(1):e0317560. doi: 10.1371/journal.pone.0317560 (PMC11759359; doi:10.1371/journal.pone.0317560)
Supplement: S1 Dataset — (DOCX) [file pone.0317560.s004.docx]

**1.Retear rate**


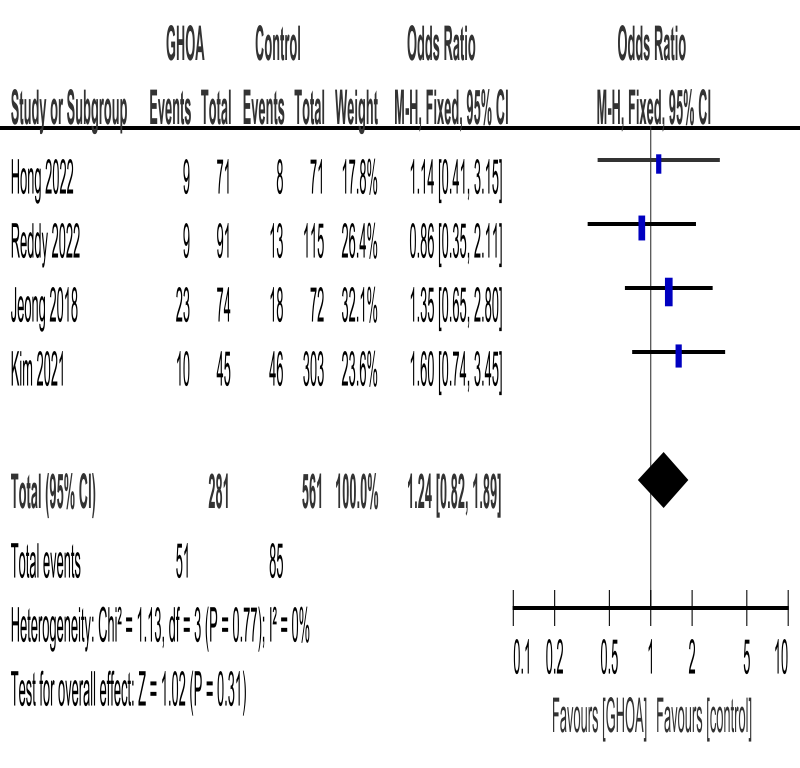


The incidence of rotator cuff retear after surgery was similar in both the case group and the control group with rotator cuff tear and GHOA (OR: 1.24; 95% CI 0.82-1.89; P=0.31).

**2.VAS**


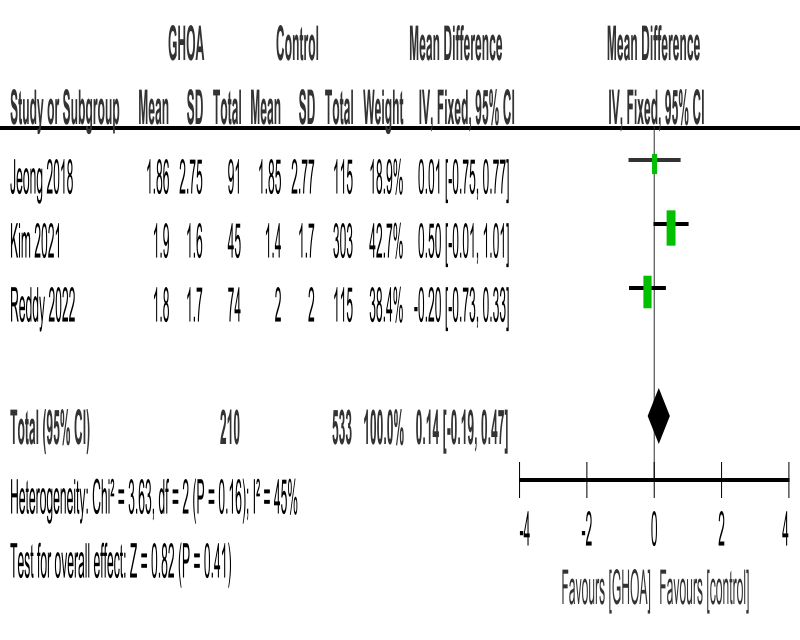


During the span of at least one year of subsequent observation, there was not a statistical difference in the VAS scores of the control group and the case group with rotator cuff injury with GHOA (MD: 0.14; 95% CI -0.19-0.47; P=0.41)

**3.ASES**


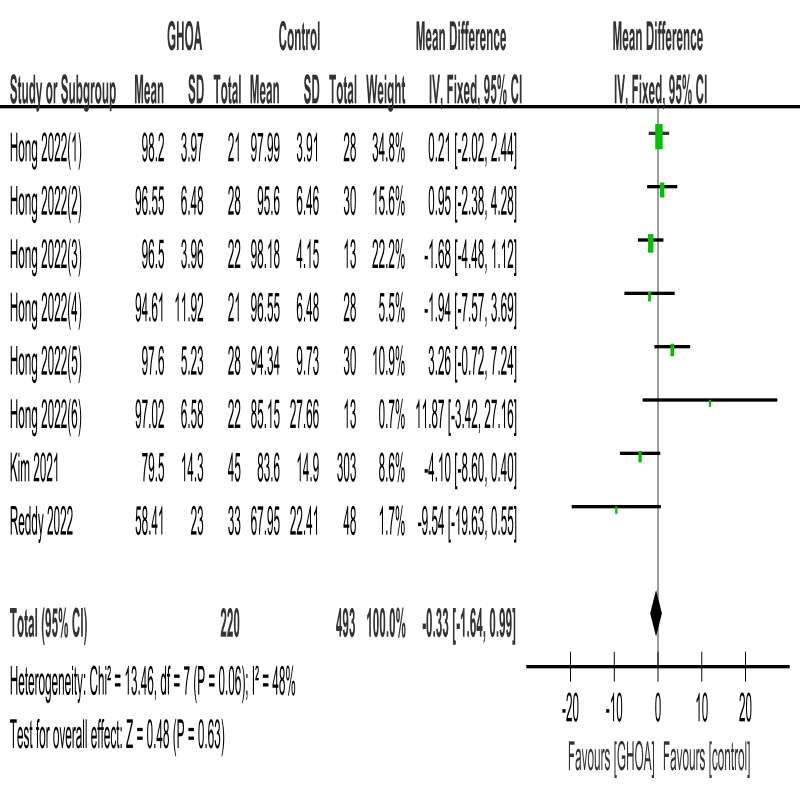


According to the findings of a meta-analysis using a fixed-effects model, there was not a statistically significant difference in the postoperative ASES ratings of patients who had rotator cuff tears and GHOA and those who were in the control group. (MD: -0.33; 95% CI -1.64-0.99; P=0.63)


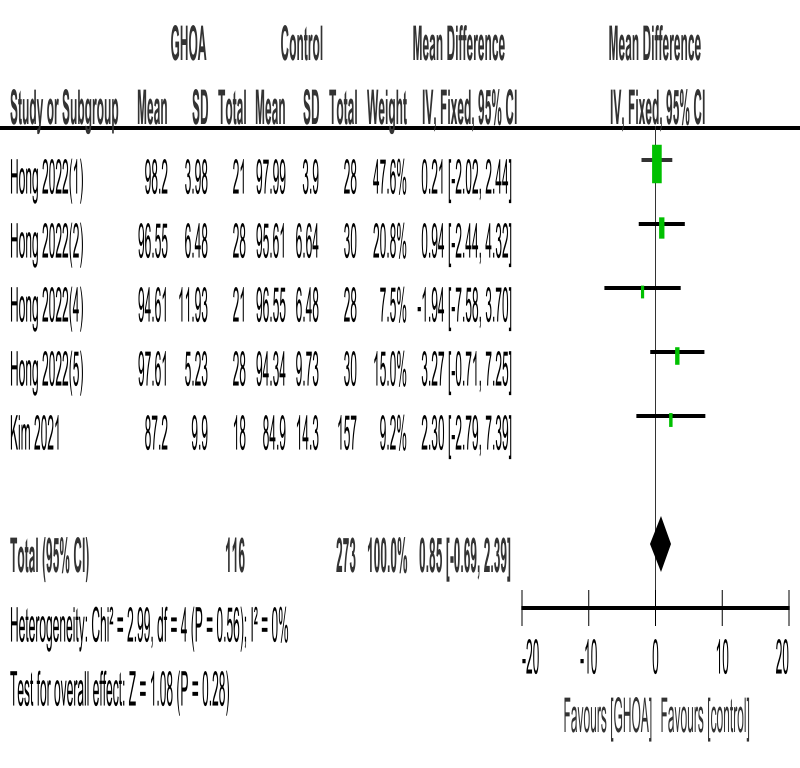


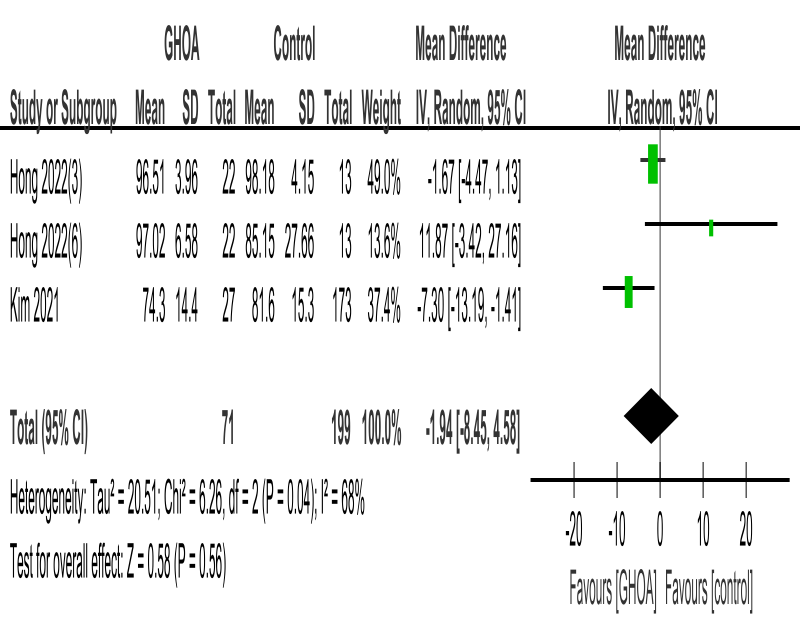


Given the varying degrees of rotator cuff tears, a subgroup analysis was conducted in this study. Participants were categorized into two groups based on the size of their tears: small to moderate tears (MD: 0.85; 95%CI -0.65-2.39; P=0.28) and large to massive tears (MD: -1.94; 95% CI -8.45-4.58; P=0.56).

**4.****Constant score**


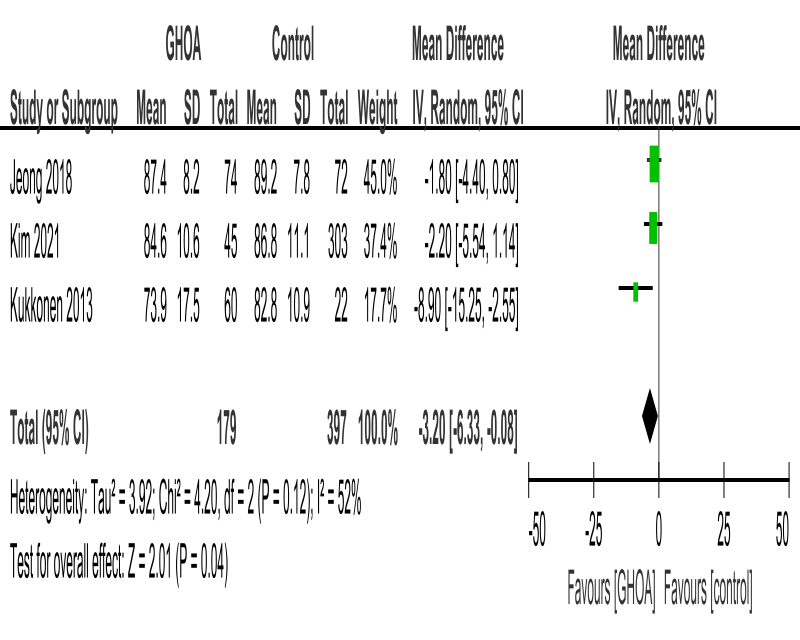


It was found that shoulder cuff repair can lead to improvements in shoulder joint function among patients with GHOA (MD: -3.20; 95% CI -6.33--0.08; P=0.04).

**5.ROM**


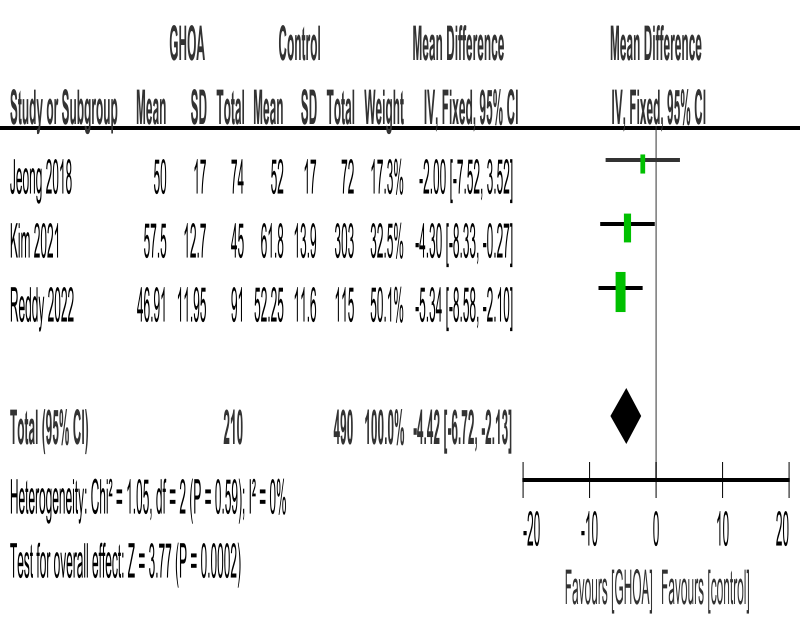


According to the findings of a meta-analysis that used a fixed effect model, there was a statistically significant difference in postoperative FF between the group of patients with rotator cuff tears who had GHOA and the control group (MD: -4.22; 95% CI -8.28--0.15; P=0.04)


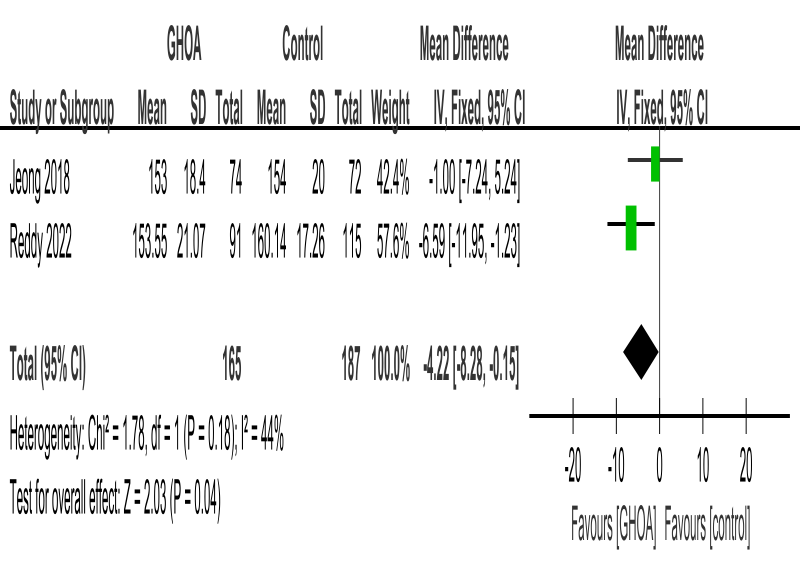


The included studies had low heterogeneity using a fixed-effect model (*I^2^*=0). Rotator cuff tear with GHOA was significantly correlated with ER (MD: -4.42; 95% CI -6.72--2.13; P=0.0002).


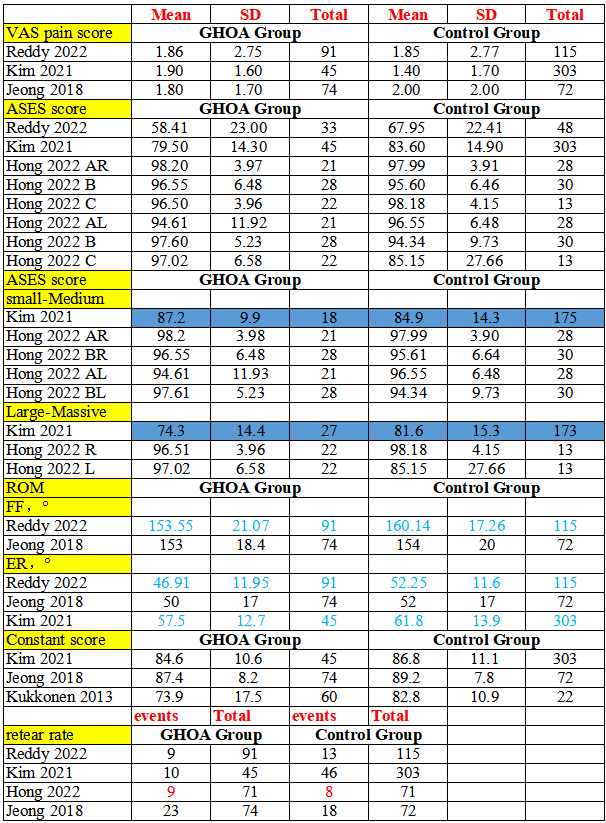


All the data in the above content comes from the 5 literatures finally included, and the 9 graphs included in this article are directly derived from the analysis of RevMan software.

1. **Kim DH**, Min SG, Lee HS, Lee HJ, Park KH, Chung SW, et al. Clinical outcome of rotator cuff repair in patients with mild to moderate glenohumeral osteoarthritis. Knee Surg Sports Traumatol Arthrosc. 2021;29(3):998-1005. doi: 10.1007/s00167-020-06307-8. PMID: 33095332
2. **Hong IS**, Rao AJ, CarlLee TL, Meade JD, Hurwit DJ, Scarola G, et al. Outcomes after arthroscopic repair of rotator cuff tears in the setting of mild to moderate glenohumeral osteoarthritis. World J Orthop. 2022;13(7):631-43. doi: 10.5312/wjo.v13.i7.631. PMID: 36051376.
3. **Jeong HY**, Jeon YS, Lee DK, Rhee YG. Rotator cuff tear with early osteoarthritis: how does it affect clinical outcome after large to massive rotator cuff repair? J Shoulder Elbow Surg. 2019;28(2):237-43. doi: 10.1016/j.jse.2018.07.022. PMID: 30337266.
4. **Reddy RP**, Solomon DA, Hughes JD, Lesniak BP, Lin A. Clinical outcomes of rotator cuff repair in patients with concomitant glenohumeral osteoarthritis. J Shoulder Elbow Surg. 2022;31(6s):S25-s33. doi: 10.1016/j.jse.2021.11.010. PMID: 34968696.
5. **Kukkonen J**, Joukainen A, Lehtinen J, Aärimaa V. The effect of glenohumeral osteoarthritis on the outcome of isolated operatively treated supraspinatus tears. J Orthop Sci. 2013;18(3):405-9. doi: 10.1007/s00776-013-0369-2. PMID: 23512014.

**Table 3 Quality assessment of included studies**

| References | Clearly stated aim | Inclusion of consecutive patient | Prospective collection of data | Endpoints appropriate for aim | Unbiased assessment of endpoints | Follow-up period appropriate follow-up period | Lost to follow-up < 5% | Prospective calculation of study size | Adequate control group | Contem-porary groups | Baseline equivalence of groups | Adequate statistical analysis | Total score |
| --- | --- | --- | --- | --- | --- | --- | --- | --- | --- | --- | --- | --- | --- |
| Reddy 2022 | 2 | 1 | 1 | 2 | 0 | 2 | 0 | 0 | 2 | 2 | 2 | 2 | 16 |
| Hong 2022 | 2 | 2 | 0 | 2 | 0 | 2 | 0 | 0 | 2 | 2 | 2 | 2 | 16 |
| Kim 2021 | 2 | 2 | 2 | 2 | 0 | 2 | 0 | 0 | 2 | 2 | 2 | 2 | 18 |
| Jeong 2018 | 2 | 2 | 2 | 2 | 0 | 2 | 2 | 0 | 2 | 2 | 2 | 2 | 20 |
| Kukkonen 2013 | 2 | 2 | 2 | 2 | 0 | 2 | 0 | 0 | 2 | 2 | 0 | 2 | 16 |

Of the 5 articles included, 4 articles were retrospective comparative study classified as Level III evidence and and1 was Level II retrospective comparative study. Based on the MINORS criteria (Table 3), the mean study quality score was 17.2 ± 1.6. These results indicated that the literature included in the study was good, which enhances the credibility of the conclusions drawn from our meta-analysis.

**Table 4 Overall evidence quality according to the** **Grading of Recommendations Assessment, Development and Evaluation (GRADE) approach**

|  | Number of studies | Study design | Certainty assessment | | | | | Effect of estimate  OR/(S)MD(95%CI) | Certainty |
| --- | --- | --- | --- | --- | --- | --- | --- | --- | --- |
|  |  |  | Risk of bias | Inconsistency | Indirectness | Imprecision | Publication bias |  |  |
| Retear rate | 4 | Observational studies | Not serious | Not serious | Not serious | Not serious | None | OR 1.24 (0.82 to 1.89) | ⨁⨁⨁◯  Moderate |
| VAS score | 3 | Observational studies | Not serious | Not serious | Not serious | Not serious | None | MD 0.14 higher (0.19 lower to 0.47 higher) | ⨁⨁⨁◯  Moderate |
| ASES | 8 | Observational studies | Not serious | Not serious | Not serious | Not serious | None | MD 0.33 lower (1.64 lower to 0.99 higher) | ⨁⨁⨁◯  Moderate |
| ASES small-Medium | 5 | Observational studies | Not serious | Not serious | Not serious | Not serious | None | MD 0.85 higher (0.69 lower to 2.39 higher) | ⨁⨁⨁◯  Moderate |
| ASES Large-Massive | 3 | Observational studies | Not serious | Not serious | Not serious | Not serious | None | MD 1.94 lower (8.45 lower to 4.58 higher) | ⨁⨁⨁◯  Moderate |
| Constant score | 3 | Observational studies | Not serious | Not serious | Not serious | Not serious | None | MD 3.2 lower (6.33 lower to 0.08 lower) | ⨁⨁⨁◯  Moderate |
| ROM(FF) | 2 | Observational studies | Not serious | Not serious | Not serious | Not serious | None | MD 4.22 lower (8.28 lower to 0.15 lower) | ⨁⨁⨁◯  Moderate |
| ROM(ER) | 3 | Observational studies | Not serious | Not serious | Not serious | Not serious | None | MD 4.42 lower (6.72 lower to 2.13 lower) | ⨁⨁⨁◯  Moderate |

According to the GRADE approach, the retear rate, VAS, ASES score, Constant score, and range of motion in FF and ER were graded as moderate(Table 4).
